# Endobronchial blocker and extracorporeal membrane oxygenation use for lung abscess complicated by a bronchopleural fistula in a pediatric patient: a case report and literature review

**DOI:** 10.3389/fped.2025.1604298

**Published:** 2025-06-19

**Authors:** Song Chen, Siwei Lu, Yuelin Sun, Wenlei Wang, Yingfu Chen, Jing Li, Chengjun Liu

**Affiliations:** Department of Critical Care Medicine, Children's Hospital of Chongqing Medical University, National Clinical Research Center for Child Health and Disorders, Ministry of Education Key Laboratory of Child Development and Disorders, Chongqing Key Laboratory of Pediatric Metabolism and Inflammatory Diseases, Chongqing, China

**Keywords:** lung abscess, bronchopleural fistula, endobronchial blocker, extracorporeal membrane oxygenation, case report

## Abstract

**Background:**

A lung abscess is a thick-walled cavity containing purulent material that results from pulmonary infection. It is an uncommon condition that can occur at any age. Bronchopleural fistula (BPF) is a severe complication with a poor prognosis that may arise with the progression of the condition or as a result of treatment.

**Case presentation:**

We describe a case of lung abscess complicated by a BPF and septic shock in a 7-year-old girl. A chest tube was inserted and venoarterial extracorporeal membrane oxygenation (ECMO) was emergently used. To selectively block the BPF, an endobronchial blocker was placed in the right intermediate bronchus under bronchoscopic guidance. This approach allowed the BPF to heal, enabled the recruitment of the other lung, and obstructed the purulent fluid. The patient recovered and was discharged after 70 days of treatment.

**Conclusions:**

This case demonstrates that combined endobronchial blocker and ECMO can be an effective approach for patients with lung abscesses and BPFs (especially those aged < 8 years) when the adjustment of conventional therapy is unsuccessful.

## 1 Introduction

A lung abscess is a thick-walled cavity containing purulent material that results from pulmonary infection. It is an uncommon condition that can occur at any age (1, 2). Based on the presence of pre-existing conditions, lung abscesses are classified as primary (occurring in previously healthy individuals) and secondary (occurring in individuals with underlying lung abnormalities) (1). These abscesses cause significant morbidity and have a mortality rate of 10%–20% in adults; in children, the mortality rate is significantly lower (1–3).

Conservative management with systemic antibiotics is the standard treatment for lung abscess. Chest tube drainage and surgery are options in cases of treatment failure or large (diameter > 6 cm) abscesses (4–6). Bronchopleural fistula (BPF) is a severe complication that may arise with the progression of the condition or as a result of treatment (drainage tube insertion) (7–9). Defined as a connection between the pleural space and bronchus causing > 24 h air leakage, BPF occurs primarily in patients who undergo lung resection (7, 10). Its prognosis is poor, with mortality rates of 67% in patients requiring mechanical ventilation and 94% in cases of major air leakage (>5 ml/kg) (7, 8).

Here, we report a case of lung abscess in a previously healthy girl, which was complicated by BPF and septic shock. It was treated successfully with combined endobronchial blocker administration and extracorporeal membrane oxygenation (ECMO).

## 2 Case presentation

A 7-year-old girl (weighing 22 kg) presented to the Emergency Department (ED) with a 20-day history of fever and non-productive cough. Twenty days previously, she had been diagnosed with *Mycoplasma pneumoniae* pneumonia, which was treated sequentially with azithromycin for 4 days, doxycycline for 3 days, and levofloxacin for 9 days. With this treatment, the patient's fever had subsided and her cough had been briefly alleviated, but her fever had returned with no worsening of the cough 3 days prior to presentation. Blood testing performed in the ED showed a white blood cell count of 20.53 × 10^9^/L, neutrophil count of 14.29 × 10^9^/L, and C-reactive protein level of 196.76 mg/L. The patient was admitted to the hospital.

**Figure 1:**
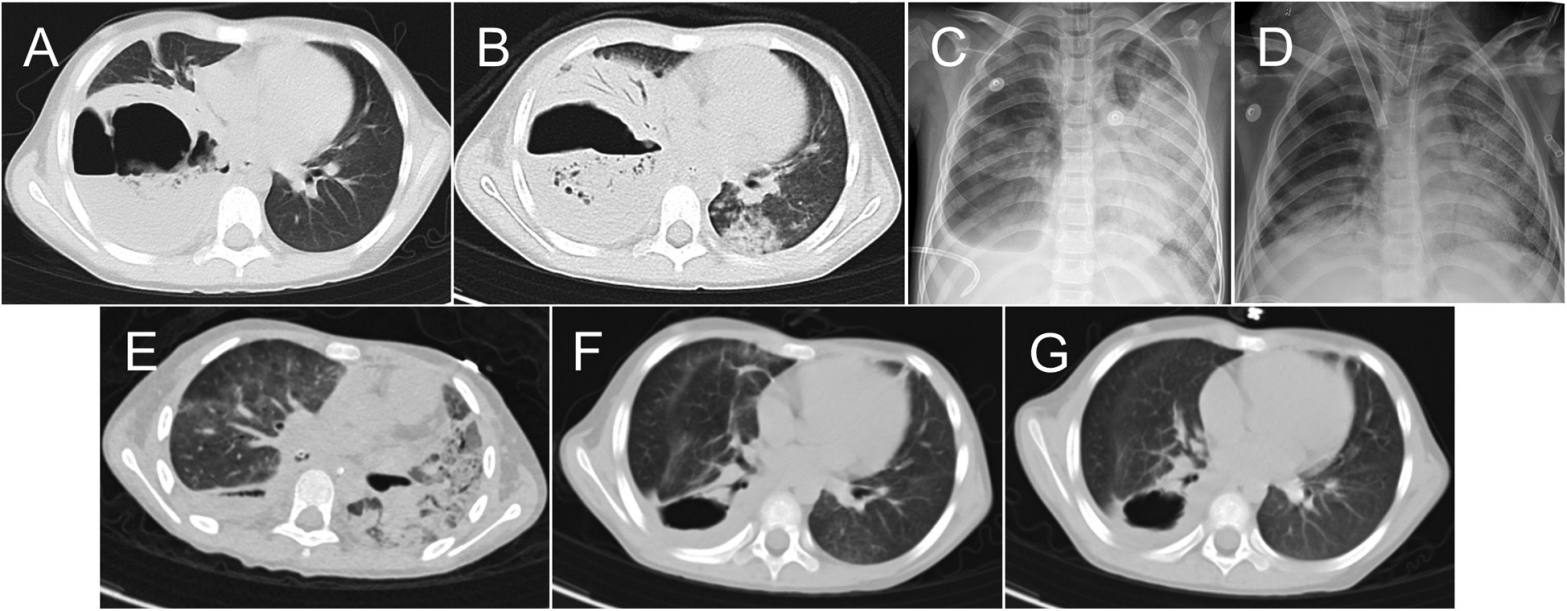
Chest images obtained during and after the patient's hospital stay. Chest computed tomography (CT) images obtained on days 1 **(A)** and 6 **(B)** of hospitalization. Chest x-rays obtained before extracorporeal membrane oxygenation initiation **(C)** and weaning **(D)** Chest CT images obtained on days 36 **(E)** and 65 **(F)** of hospitalization and 1 month after discharge **(G)**.

A chest computed tomography (CT) examination performed after admission showed a large cavity in the right inferior lobe of the lung with a pronounced gas-fluid level (Figure 1A), leading to the diagnosis of lung abscess. Polymerase chain reaction (PCR) analysis of the patient's sputum revealed *M. pneumoniae* (2.28 × 10^7^) and A2063G/A2064G positivity. As the most common pathogens causing lung abscess are *Streptococcus pneumoniae* and *Staphylococcus aureus*, a combination of levofloxacin (10 mg/kg/day) and vancomycin (60 mg/kg/day) was administered.

Six days after admission, the patient's condition declined, with tachypnea, moaning, and pallor. She was transferred to the pediatric intensive care unit (PICU), where invasive mechanical ventilation was initiated. A contrast-enhanced chest CT examination showed no significant change to the large cavity, but additional lesions in other parts of the lungs (Figure 1B). Bronchoscopy revealed large amounts of yellowish-white secretion in the bronchus, suggesting that the lung abscess had ruptured.

Seven days after admission, the patient experienced worsening hypoxemia [partial pressure of oxygen (PaO_2_) = 53.7 mmHg, fraction of inspired oxygen (FiO_2_) = 1.0] and hypercarbia [partial pressure of carbon dioxide = 68.7 mmHg]. A chest x-ray demonstrated significantly increased opacities, accompanied by consolidation in the left lung (Figure 1C). Bronchoalveolar lavage was urgently performed, but the patient's oxygenation continued to worsen (PaO_2_ = 36.4 mmHg, FiO_2_ = 1.0) and she exhibited hypotension (blood pressure = 58/34 mmHg with 0.1 ug/kg/min noradrenaline). We decided to emergently perform venoarterial extracorporeal membrane oxygenation (VA-ECMO). Considering the size of the lung abscess and the risk of bleeding with the performance of percutaneous transthoracic tube drainage after ECMO initiation, a chest tube was inserted into the right chest before ECMO administration, producing a gush of air followed by continuous air leakage and purulent fluid drainage. Fourteen- and 19-Fr heparin-coated cannulas (Bio-Medicus; Medtronic Inc., Minneapolis, MN, USA) were implanted surgically via the right common carotid artery and internal jugular vein. The patient's antibiotic treatment was adjusted (imipenem/cilastatin, 15 mg/kg every 6 h; linezolid, 10 mg/kg every 8 h; voriconazole, two loading doses of 9 mg/kg every 12 h, maintenance doses of 8 mg/kg every 12 h; and doxycycline, 2 mg/kg every 12 h).

**Figure 2:**
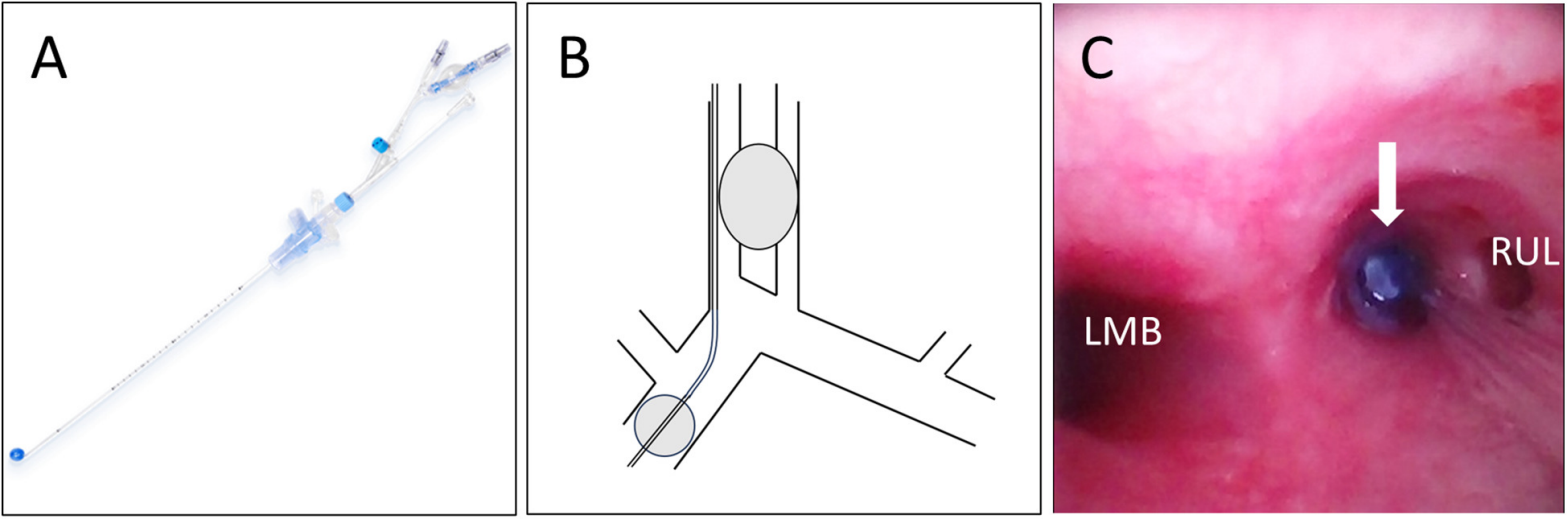
Endobronchial blocker placement. **(A)** An endobronchial blocker. Diagram **(B)** and bronchoscopic image **(C)** of the endobronchial blocker occluding the right intermediate bronchus, which resolved the air leakage through the bronchopleural fistula. LMB, left main-stem bronchus; RUL, right upper lobe.

Lung-protective ventilation was considered [Initiation ventilator settings: Tidal volumes 4–6 ml/kg, FiO_2_ 30%, post end-expiratory pressure (PEEP) 5 cmH_2_O, Respiratory rate 10 breaths per minute], as most of the tidal volume was leaking continuously through the chest tube and gas exchange could not be maintained in either lung. On bronchoscopy, yellowish-white secretions could be aspirated continuously from the inferior lobe of the right lung (RB9), indicating that a BPF had developed in that location. An endobronchial blocker (auto-inflation tube, external diameter = 2.3 mm; Tappa Medical Technology Co., Hangzhou, China) was guided into the right intermediate bronchus through the outside of the endotracheal tube by bronchoscopy (Figure 2). The air leakage decreased significantly as soon as the endobronchial balloon occupied the right intermediate bronchus. We adjusted the balloon filling degree to allow a small amount of air to leak from the fistula, discharging the purulent fluid, and maintained PEEP at 8–12 cmH_2_O with the help of the endobronchial blocker. Bronchoalveolar lavage was performed repeatedly over the subsequent days to eliminate the purulent fluid from the bronchus.

Culture of the purulent fluid yielded *Pseudomonas aeruginosa*, and next-generation sequencing of the bronchoalveolar lavage fluid revealed *P. aeruginosa* and *M. pneumoniae*. According to these pathogen results, we stopped the voriconazole.

After 16 days of support, the patient's status improved; Figure 1D is a chest x-ray obtained before she was weaned from ECMO. On day 24 of hospitalization, the patient was successfully weaned from ECMO and decannulated. High-frequency oscillatory ventilation was used for 5 days, followed by conventional mechanical ventilation. A chest CT examination performed on day 36 showed extensive parenchyma and interstitial lesions in the bilateral lungs (Figure 1E). A systemic glucocorticoid (methylprednisolone sodium succinate, 1.5 mg/kg every 12 h) was administrated. An abdominal CT examination revealed liver and spleen abscesses. Considering the possibility of anaerobic infection, we added metronidazole (7.5 mg/kg every 8 h) to the patient's treatment regimen. As air still leaked continuously from the chest tube when the endobronchial blocker was released, blocker use was continued until day 41 of hospitalization, when we observed significantly decreased air leakage. At that time, we adjusted the chest drainage suction level from 0 to −10 cmH_2_O to aid purulent fluid drainage. On day 46 of hospitalization, the patient was weaned from ventilation. On day 56, no bubbling in the water seal of the chest drainage was seen and the chest tube was removed. Figure 1F is a chest CT image acquired on day 65 of hospitalization, when the patient was discharged from the PICU. The patient was discharged from the hospital without surgical intervention on day 70. Chest CT re-examination was performed 1 month after discharge (Figure 1G), and the girl returned to school with no symptom.

## 3 Discussion

In the case reported here, a lung abscess complicated by a BPF in a pediatric patient was treated successfully with combined endobronchial blocker and ECMO use. This report is the first to describe the use of this approach for lung isolation to manage these conditions in a patient aged < 8 years.

ECMO is an effective supportive method applied to patients with refractory respiratory failure and BPFs (7). It enables the control of infection with systemic antibiotics, ventilator-based management with reduced parameters to allow the lungs to rest, and BPF healing and closure. A few case reports and series describing the use of ECMO in BPF treatment have been published (11, 12). While most cases can be managed with venovenous ECMO (VV-ECMO), VA-ECMO was utilized in the current case. We selected VA-ECMO for two reasons: the unavailability of double-lumen cannulas in China limited VV access via the right internal jugular vein, and the patient's femoral vein was insufficiently sized to provide the target blood flow. Daoud et al. (8) reported the use of VA-ECMO for five patients with BPF and acute lung injury after thoracic surgery. Lung-protective ventilation was employed after ECMO initiation, and three of the patients recovered.

Ventilator management is crucial for patients with BPF. Conventional ventilation strategies include decreasing peak-inspiratory pressure by using lower tidal volumes, lowering PEEP, decreasing the inspiratory time, and decreasing the respiratory rate. High-frequency ventilation has also been evaluated for managing refractory air leak (7, 13). During ECMO support, ventilator pressure can be reduced or turned off altogether for hours or days until the leak seals. These approaches are suitable for patients with BPFs secondary to pneumonectomy or thoracic trauma which without severe infection. Grant et al. (13) shared successful utilization of ECMO with lung protective strategies to three traumatic BPF.

In the current case, the patient had a BPF secondary to a right lung abscess. Copious amounts of purulent fluid flooded into the left lung, resulting in the left lung consolidation. Sufficient drainage and protection of the rest lung from contamination was critical. Conventional ventilation strategies and high-frequency ventilation could not obstruct and drain the purulent fluid. Lung isolation can eliminate air leakage through a BPF, obstruct the purulent fluid, and improve gas exchange in the contralateral lung (7). It is most commonly performed using a double-lumen endotracheal tube. Garlick et al. (14) reported the successful treatment of trauma (lung contusion with prominent pneumatoceles that evolved into a BPF with persistent left-lung air leakage) in a 16-year-old boy with differential lung ventilation using a double-lumen tube and VV-ECMO. However, even the smallest double-lumen tube is too large for patients aged < 8 years. In addition, double-lumen tubes are not convenient for the performance of repeat bronchoscopy. Endobronchial intubation is the simplest lung isolation technique, but also impedes repeat bronchoscopy. Endobronchial blockers have been used in pediatric patients in the operating room (15). In the current case, the endobronchial blocker obstructed purulent fluid, preventing infection of the rest lung, and facilitated bronchoscopy performance. It also promoted collapse of the diseased lung and BPF healing. Simultaneously, with the blocker enabled adequate ventilation of the consolidated lung and recruitment.

The timing of ECMO weaning also deserving discussion. Maintaining ECMO support until BPF healing may represent a safer process. All three patients in Grant's report were weaned from ECMO after BPF resolved (11). In our case, we weaned ECMO and decannulated prior to BPF closure, with the BPF persisting for 30 more days. Our weaning decision was based on the following considerations. Unlike the patients reported by Grant et al, the fistula in this case was secondary to severe infection, requiring more time for healing. Prolonged duration of ECMO increases the risk of adverse effects. Before weaning, the patient was suffering cannulation site bleeding and right thumb ischemia. Concurrently, effect of the circuit was diminishing. Most importantly, the patient's left lung got significantly improved. Based on these factors, we performed a trial off, and it was successful. Consequently, we decided to wean and decannulate. With the endobronchial blocker in place, the patient tolerated higher ventilator settings post-ECMO.

## 4 Conclusions

With this case, we demonstrate that combined endobronchial blocker and ECMO use can be an effective approach for patients with lung abscesses and BPFs when the adjustment of conventional therapy is unsuccessful. It is especially suitable for patients aged < 8 years.

## Data Availability

The raw data supporting the conclusions of this article will be made available by the authors, without undue reservation.
